# Increased Oxidative Burden Associated with Traffic Component of Ambient Particulate Matter at Roadside and Urban Background Schools Sites in London

**DOI:** 10.1371/journal.pone.0021961

**Published:** 2011-07-27

**Authors:** Krystal J. Godri, Roy M. Harrison, Tim Evans, Timothy Baker, Christina Dunster, Ian S. Mudway, Frank J. Kelly

**Affiliations:** 1 Division of Environmental Health & Risk Management, School of Geography, Earth & Environmental Sciences, University of Birmingham, Edgbaston, Birmingham, United Kingdom; 2 MRC-HPA Centre for Environmental Health, School of Biomedical and Health Sciences, King's College London, London, United Kingdom; University of Liverpool, United Kingdom

## Abstract

As the incidence of respiratory and allergic symptoms has been reported to be increased in children attending schools in close proximity to busy roads, it was hypothesised that PM from roadside schools would display enhanced oxidative potential (OP). Two consecutive one-week air quality monitoring campaigns were conducted at seven school sampling sites, reflecting roadside and urban background in London. Chemical characteristics of size fractionated particulate matter (PM) samples were related to the capacity to drive biological oxidation reactions in a synthetic respiratory tract lining fluid. Contrary to hypothesised contrasts in particulate OP between school site types, no robust size-fractionated differences in OP were identified due high temporal variability in concentrations of PM components over the one-week sampling campaigns. For OP assessed both by ascorbate (OP^AA^ m^−3^) and glutathione (OP^GSH^ m^−3^) depletion, the highest OP per cubic metre of air was in the largest size fraction, PM_1.9–10.2_. However, when expressed per unit mass of particles OP^AA^ µg^−1^ showed no significant dependence upon particle size, while OP^GSH^ µg^−1^ had a tendency to increase with increasing particle size, paralleling increased concentrations of Fe, Ba and Cu. The two OP metrics were not significantly correlated with one another, suggesting that the glutathione and ascorbate depletion assays respond to different components of the particles. Ascorbate depletion per unit mass did not show the same dependence as for GSH and it is possible that other trace metals (Zn, Ni, V) or organic components which are enriched in the finer particle fractions, or the greater surface area of smaller particles, counter-balance the redox activity of Fe, Ba and Cu in the coarse particles. Further work with longer-term sampling and a larger suite of analytes is advised in order to better elucidate the determinants of oxidative potential, and to fuller explore the contrasts between site types.

## Introduction

Children living or attending schools near busy roads have been reported to suffer from increased rates of respiratory problems, as well as impaired lung growth [1]–[5]. Consistent with a causal relationship between traffic emissions and impaired respiratory health Wjst et al. [6] demonstrated an inverse relationship between children's lung function with traffic density on the road servicing their school. In addition to traffic density, fleet composition also appears important, with evidence that children attending schools less than 100 m from roads carrying a high proportion of diesel vehicles suffer from not only reduced lung function, but also increased chronic respiratory symptoms, including cough, wheeze, and rhinitis [7].

Schools located near busy roads will experience increments in primary traffic emissions including nitrogen oxides, carbon monoxide and particulate matter (PM). Studies referenced above investigating the respiratory responses to traffic exposure used traffic counts and proximity to roads as a proxy measure of primary vehicular emission exposure. It is however likely that a roadside source profile will provide even greater contrasts in PM composition relative to that observed at urban background or rural locations; appreciating these compositional contrasts will prove important in understanding the basis of the health effects observed in high traffic micro-environments. In this study two weekly sampling campaigns were performed at seven sites within the London Air Quality Network (LAQN) to examine composition and toxicity contrasts in gaseous and size-fractionated particulate pollutant concentrations at primary schools located near major roads and at urban/suburban background locations.

Whilst the association between adverse respiratory health and exposure to elevated concentrations of PM is robust, the strength of this relationship differs markedly between studies [8]–[10]. This heterogeneity likely reflects in part the use of bulk PM mass concentration as an exposure metric, which is insensitive to variation in physical and chemical characteristics, attributable to varying source contributions at a given location. Much attention has therefore been directed towards identifying components, or characteristics of PM that could be used to provide more precise metrics of their potential health impacts. As PM toxicity likely reflects the sum of multiple toxic components approaches focusing on single factors (e.g. particle size, chemical components) are unlikely to be fruitful. A measure of PM toxicity that integrates composition across size-fractions would seem a more logical strategy, with the long-term aim of establishing a ‘biologically-active’ PM metric for ambient air. PM oxidative potential (OP) has been proposed as such a metric, with numerous toxicological studies documenting PM-induced oxidative stress within the lung [11]–[13]. Oxidative potential, as measured in a cell free model, either by EPR, antioxidant depletion or DTT oxidation, reflects the integrated sum of its content of redox active components. ROS can also clearly be formed through the interaction of non-redox active components with tissues in vivo, though the relative balance between intrinsic and latent oxidative activities still needs to be accurately quantified, which is almost impossible in cell models which are already under a state of continuous oxidative stress [14]–[15]. This metric was examined in the present study to identify individual PM fractions and components that contribute significantly to the observed oxidative activity of ambient PM.

## Materials and Methods

### Sampling site description

**Figure 1:**
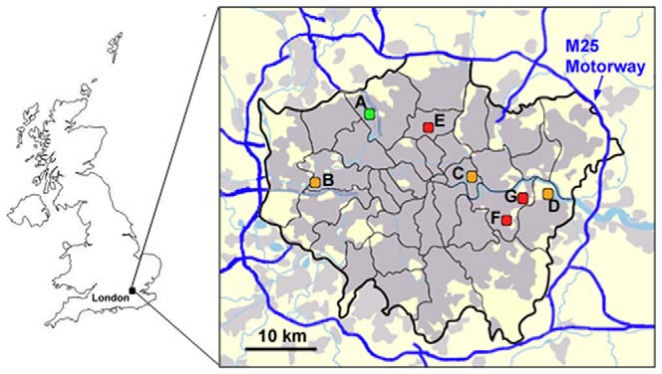
Primary school sampling sites in the Greater London Urban Area. Site A represents the suburban site. The urban background and roadside are indicated by sites B–D and sites E–G, respectively. The M25 motorway encircles the city and drawn in blue while the black lines mark the boroughs of London.

Seven London primary school sites were selected to represent a wide geographical area (northwest to southeast London) (Figure 1). School sampling locations were classified as suburban (residential area on the outskirts of a town or city, Site A), urban background (Sites B–D), or roadside (sampling inlet between 1 and 5 m from the kerbside, Sites E–G). The sampling inlet height was positioned to 2 m above ground level at each site with the exception of Urban Background Site C which was located on the roof-top of a three-storey school building. Site selection was limited to primary schools which housed a LAQN monitoring station on its grounds or in proximity.

### Sampling instrumentation

All LAQN sites selected were equipped with a Tapered Element Oscillating Microbalance (TEOM) with a 50°C heated inlet or R&P Filter Dynamics Measurement System (FDMS). Continuous mass concentrations of PM with an aerodynamic diameter of less than 10 (PM_10_) and 2.5 µm (PM_2.5_) were measured with 15 minute resolution by the TEOM or FDMS. To account for semi-volatile particulate matter losses by TEOM measurements, the volatile correction model (VCM) was applied, as recommended for comparison of TEOM PM_10_ measurements with the European Directive [16]. Continuous 15 minute resolved gaseous nitrogen oxide (NO) and nitrogen dioxide (NO_2_) measurements were taken at each LAQN site. This was further complemented with wind direction averaged at the same sampling interval at select Urban Background Sites B–D and Roadside Sites F and G.

The pre-existing measurement infra-structure at each LAQN monitoring stations was augmented on a rolling basis for a period of 14 days during November 2007 and March 2008. Added instrumentation included a Micro-Orifice Uniform Deposit Impactor (MOUDI) and a Condensation Particle Counter (TSI CPC, Model 3022A). The CPC particle number concentration (PNC) measurements were recorded continuously with 15 minute resolution.

The total 14-day campaign at each site was subdivided into two 7-day MOUDI filter sampling periods. The MOUDI, operated at 30 L min^−1^, sampling size-fractions of PM with aerodynamic diameters less than 0.2 µm (PM_0.2_), between 0.2 and 1 µm (PM_0.2–1_), between 1 and 1.9 µm (PM_1–1.9_) and between 1.9 and 10.2 µm (PM_1.9–10.2_). All Teflon MOUDI filters were weighed before and after air sampling using a Sartorius model MC5 microbalance to determine the PM mass. Filters were equilibrated for 24 hours prior to weighing in a room with humidity (45 – 55%) and temperature (20±2°C) controls. To eliminate static electricity effects on filter weights, an ionising blower and α-particle source were employed. Following sampling, each MOUDI filter was halved for subsequent compositional and OP analysis.

The equivalence of PM_10_ mass concentrations derived from MOUDI filter samples and co-located semi-continuous TEOM measurements was evaluated. The latter was averaged to a seven-day basis comparable to the MOUDI filters' resolution. At least 75% of the fifteen minute TEOM measurements were required for weekly averages to be considered valid, excluding Sites F and G from this instrument inter-comparison. For Sites A–E, VCM corrected TEOM mass concentrations were greater than measurements given by the corresponding filter data (Table 1). The Bland-Altman method was considered to evaluate the equivalence between the two instrument measurements; the sampling methods consistently provided similar measurements (p = 0.0014). The mean measurement difference was −7.1 between the MOUDI and VCM corrected TEOM with 95% limits of agreement (−16.2, 2.0) containing all the difference scores. Temporal agreement was further investigated between the two instrument measurements (r^2^ = 0.86, p<0.0001, n = 10); despite the instrument agreement suggested by the Bland-Altman analysis, the linear regression slope (0.51 (µg m^−3^)_MOUDI_/(µg m^−3^)_VCM TEOM_) between MOUDI and VCM TEOM PM_10_ mass concentrations suggested a systematic bias. The non-unit MOUDI/VCM TEOM ratio which is consistent across all considered school sites is representative of semi-volatile constituent losses from the filter: the discrepancy between the two PM mass sampling methods was improved when raw TEOM concentrations were considered characterised by a linear regression slope of 0.83 (µg m^−3^)_MOUDI_/(µg m^−3^)_Raw TEOM_). Semi-volatile losses from the MOUDI Teflon filters are acknowledged as a weakness of the study's sampling setup, but cannot be avoided when size-fractionated sampling is required.

**Table 1:** Summary of temperature (mean), nitrogen oxides (mean), PM number concentrations (PNC; median), VCM corrected TEOM and MOUDI PM_10_ mass (mean) at primary school sampling sites.

SchoolID	SiteType	Sampling Dates	Temp (°C)	NO/NO_2_	NO_x_ (µg m^−3^)(as NO_2_)	PNC (particlescm^−3^)	Raw TEOM PM_10_(µg m^−3^)	VCM TEOM PM_10_(µg m^−3^)	MOUDI PM_10_(µg m^−3^)
A	SU	Feb 22–29, 2008	**NA**	**0.30** (0.05; 1.30)	**62.3** (13.4; 265.5)	**8,147** (3,437; 28,263)	**14.0** (4.5; 27.3)	**17.1** (6.8; 30.4)	**13.2**
		Feb 29–Mar 6, 2008	**NA**	**0.17** (0.00; 0.53)	**33.4** (3.7; 122.7)	**6,428** (1,270; 17,513)	**13.5** (3.0; 23.8)	**14.4** (4.3; 26.5)	**11.6**
B	UB	Jan 30–Feb 6, 2008	**6.8** (1.7; 11.7)	**0.09** (0.00; 0.36)	**35.9** (7.5; 96.2)	**16,249** (3,268; 86,742)	**14.7** (4.1; 26.1)	**14.0** (2.0; 25.8)	**11.6**
		Jan 6–14, 2008	**7.5** (1.6; 16.0)	**0.90** (0.03; 2.89)	**184.8** (26.8; 514.8)	**25,447** (5,670; 51,984)	**27.7** (11.1; 52.8)	**36.5** (16.2; 68.0)	**25.2**
C	UB	Jan 9–16, 2008	**9.1** (4.8; 11.4)	**0.11** (0.00; 0.32)	**36.0** (8.3; 87.3)	**14,110** (3,271; 29,446)	**14.3** (2.0; 26.6)	**14.4** (3.3; 26.1)	**12.2**
		Jan 16–25, 2008	**7.6** (4.8; 11.7)	**0.16** (0.00; 0.76)	**50.2** (6.8; 176.4)	**8,796** (2,323; 33,547)	**23.9** (5.5; 75.4)	**23.9** (5.3; 78.9)	**18.0**
D	UB	Dec 12–19, 2007	**2.9** (−1.5; 5.6)	**0.99** (0.06; 3.28)	**187.5** (31.1; 672.8)	**26,559** (6,260; 71,742)	**26.1** (12.9; 47.6)	**36.8** (22.1; 62.8)	**22.0**
		Jan 3–9, 2008	**7.2** (3.5; 10.3)	**0.23** (0.03; 0.69)	**38.5** (5.2; 103.1)	**7,025** (2,118; 27,091)	**21.0** (7.5; 51.6)	**27.7** (7.0; 67.4)	**14.3**
E	RS	Mar 6–13, 2008	**8.7** (5.8; 12.7)	**0.44** (0.10; 0.96)	**67.3** (21.6; 152.4)	**13,822** (4,847; 30,435)	**19.1** (6.0; 36.4)	**19.4** (5.8; 35.7)	**12.5**
		Mar 13–19, 2008	**7.9** (2.8; 14.3)	**0.56** (0.10; 1.20)	**91.8** (23.2; 216.0)	**15,495** (5,119; 38,350)	**18.2** (8.4; 31.7)	**21.9** (10.7; 38.4)	**14.3**
F	RS	Nov 14–21, 2007	**6.2** (−0.1; 11.1)	**1.81** (0.33; 4.20)	**343.3** (24.2; 1140)	**46,004** (6,169; 159,633)	**NA**	**NA**	**24.5**
		Nov 21–28, 2007	**6.3** (−0.1; 11.1)	**0.88** (0.05; 2.56)	**147.8** (29.6 424.3)	**30,549** (8,024; 137,295)			**26.2**
G	RS	Nov 28–Dec 5, 2007	**11.4** (8.6; 15.0)	**0.58** (0.15; 1.06)	**83.6** (12.1; 231.9)	**9,779** (2,175; 33,001)	**NA**	**NA**	**12.1**
		Dec 5–12, 2007	**9.0** (2.1; 15.4)	**0.85** (0.16; 2.84)	**156.9** (12.4; 716.8)	**17,859** (2,714; 82,569)			**15.2**

The associated 5^th^ and 95^th^ percentiles are also given in brackets for semi-continuous measurements. Note: SU, sub-urban; UB: urban background; RS, roadside; NA, not available.

### Chemicals and chelex water preparation

All chemicals used were obtained from Sigma Chemical Company Ltd (Poole, UK) and were of high pressure liquid chromatography grade. For chemical composition analysis, 18.2 MΩ-cm Millipore distilled deionised water was used. All water for OP analysis was deionised and ultra-filtered using an Elga-stat filtration system and further treated with Chelex-100 resin to eliminate any background metal contamination.

### Compositional analysis

A quarter of the MOUDI filter from each impactor stage was used for water soluble inorganic ions and another quarter for total trace metal analysis. For the latter, filter segments were extracted using a dilute reverse aqua regia solution made up in distilled deionised water prior to sonication in an 80°C water bath for 1 hour [17]. The solution was allowed to cool prior to filtration through an Acrodisc Syringe Filter (Pall Acrodisc 32 mm Syringe Filter, 0.45 µM Supor Membrane). Acid extracted solutions were stored at 4°C until analysis for concentrations of ^27^Al, ^137^Ba, ^52^Cr, ^65^Cu, ^57^Fe, ^60^Ni, ^51^V, and ^66^Zn using inductively coupled plasma mass spectrometry (Aligent 7500ce with an Octopole Reaction System). All material (pipette tips, scissors, vials) that came into contact with the filters, or the filter extract solution were plastic and acid washed (2% HNO_3_) prior to use.

Filter material for water soluble cation (Na^+^, NH_4_^+^, K^+^, Mg^2+^, Ca^2+^) and anion (Cl^−^, NO_3_^−^, SO_4_^2−^, PO_4_^3−^) analysis was extracted in distilled deionised water (18.2 MΩ-cm) following mechanical shaking for 20 minutes. The resultant PM suspensions were then filtered with an Acrodisc Syringe Filter in a similar way to acid extracts. Anion species were measured on a Dionex ICS-2000 ion chromatography system eluted with electrodialytically generated (EG40) 20.5 mM KOH mobile phase. The ICS-2000 system was fitted with an anion guard (AG11-HC, 2×50 mm) and separator (AS11-HC, 2×250 mm) columns, both of which were maintained at 30°C. Sample solutions, following separation entered the ASRS-Ultra 52 mA suppressor and then a 2 mm carbonate removal device before passing through the thermally stabilised conductivity cell. A Dionex model DX100 ion chromatograph was used to measure cation species. PM extracts were then passed through a cation guard (CG12A) and analytical (CS12A) columns and finally routed into a conductivity detector (CD25A). The system was isocratically eluted with a 30 mN sulphuric acid mobile phase at a flow rate of 1.0 ml min^−1^. Each system was calibrated with multi-elemental standards prepared with working concentrations in the range 0.2 to 10 ppm.

### Assessment of oxidative potential

PM collected on MOUDI filters was extracted in methanol by vortexing (10 minutes) and probe sonication (MSE Soniprep 150) using a titanium probe at an amplitude of 5 microns for 30 seconds. The extracted PM suspension was then evaporated to dryness under a gentle stream of nitrogen prior to resuspension to 150 µg mL^−1^ using Chelex-resin treated water containing 5% methanol.

An acellular model was employed to assess the OP of the size fractionated PM samples. Antioxidant depletion from a synthetic respiratory tract lining fluid (RTLF) was measured following a four hour incubation at 37°C to uniform mass concentrations of resuspended particulate matter (50 µg mL^−1^). The RTLF chemical model was comprised of physiologically relevant concentrations (200 µM) of urate (UA), ascorbate (AA) and glutathione (GSH) adjusted to pH 7.0. Following PM-RTLF exposures, samples were centrifuged at 13,000 rpm for 60 minutes at 4°C and then antioxidant concentrations were measured.

For AA and UA measurements, 50 µL of the incubated RTLF-PM supernatant was combined with 450 µL of ice cold 5% metaphosphoric acid [18]. Reverse phase high pressure liquid chromatography with an EG&G amperometric electrochemical detector (Jones Chromatography, Hengoed, Wales) was used to simultaneously determine these antioxidant concentrations. The detector was operated with an electrode voltage of 400 mV, 5 second time constant and 500 nA cathodic output sensitivity. A 25 µL aliquot of sample solution was injected, using an auto-sampler (Model 231, Gilson), onto a analytical column (10×300 nm, 5 µm C18) and eluted with 0.2 mM K_2_HPO_4_-H_3_PO_4_ mobile phase containing 0.25 mM octanesulphonic acid (pH 2.1) at a flow rate of 1.0 mL min^−1^. AA and UA calibration curves (0–25 µM) were used to calculate sample concentrations.

The GSH concentration remaining in the RTLF after the 4 hour incubation was indirectly calculated by measuring total glutathione (GSx) and glutathione disulphide (GSSG) concentrations. These compounds were determined using the GSSG-reductase- 5,5′-dithio-bis(2-nitrobenzoic acid) assay [19]. A 16.7 µL aliquot of the incubated RTLF-PM supernatant was added to 983.3 µL of 100 mM sodium phosphate buffer (pH 7.5) containing ethylenediaminetetraacetic acid. In parallel with all sample batches, a GSSG standard curve (0 – 165 pmol/50 µL GSSG, equivalent to 0 – 330 pmol/50 µLGSx) was also run. To measure GSx concentrations, 50 µL of the sample and standard solutions were dispensed onto a microtitre plate. Once 100 µL of reaction mixture (0.15 mM 5,5′-dithio-bis(2-nitrobenzoic acid), 0.2 mM NADPH and 1 U glutathione reductase) was added to each well, the plate was immediately analysed on a plate reader (SpectraMAX 190; Molecular Devices) heated to 30°C. The total analysis period was two minutes during which time the formation rate of 5-thio-2-nitrobenzoic acid was measured at an absorbance of 405 nm. A similar protocol was employed to measure GSSG concentration. The only exception was that prior to sample combination with the reaction mixture, 5 µL of 2-vinyl pyridine was combined with 130 µL of sample and control solutions. These 2-vinyl pyridine solutions were vortexed for 5 seconds, followed by a one hour incubation period at room temperature. GSH was calculated by subtracting two times the GSSG concentration from the measured GSx concentration.

To guarantee inter-experimental standardisation, known particle and particle-free controls (positive: residual oil fly ash; negative: M120 carbon black) were also run in parallel with filter samples. Control material was resuspended similarly to ambient PM samples in Chelex water containing 5% methanol and probe sonicated to ensure the suspensions were homogeneous. Compositional details of the residual oil fly ash [18]–[23] and M120 [24], [25] control particles have been published elsewhere.

The percentage of AA and GSH depletion over the 4 hour incubation period by each PM sample was calculated relative to particle-free control run in parallel. PM OP expressed on a unit mass basis was determined by standardising the percentage loss value by the final PM mass concentration used in the RTLF assay (50 µg mL^−1^) for ascorbate (OP^AA^ µg^−1^) and reduced glutathione (OP^GSH^ µg^−1^). PM OP was also calculated on a unit volume basis by accounting for ambient PM mass concentrations to yield individual measures describing ascorbate (OP^AA^ m^−3^) and reduced glutathione (OP^GSH^ m^−3^) depletion.

### Statistical analysis

SPSS for windows (version 17) was used for statistical analyses. Comparison of PM chemical composition concentrations and OP measurements across sampling sites and PM size fractions were evaluated using one-way ANOVA with post-hoc testing using the Tukey HSD test. Variation of PM OP was evaluated using physico-chemical measurements (particle number, total trace metal and soluble inorganic ion concentrations) as explanatory variables in uni- and multi-variate linear regressions. The strength of uni-variate associations between these independent PM constituent variables and the OP metrics (AA and GSH expressed on a unit mass and volume basis) were assessed with Pearson correlations. Stepwise multi-variate linear regression analysis was also performed with a backward deletion procedure. Interactions between PM constituents were also considered and were used to form a priori assumptions regarding the covariates entered into multi-variate linear regression model. A *p*-value less than 0.05 was considered to be significant for all cases.

## Results and Discussion

### Vehicular emission related PM

Evaluation of continuous PNC, PM_10_ and gaseous (NO_x_ = NO+NO_2_) pollutant concentrations collected over each site's two week campaign period (Table 1) did not indicate any significant increments at the roadside sites compared to urban background and suburban site types. This is not surprising as ambient measurements were collected sequentially across the school locations over short durations which enabled episodic periods to skew the PM and gaseous pollutant concentration distributions. When NO_x_ and PM_10_ mass concentrations were considered for equivalent extended periods (the full academic year spanning September to June) at each of these schools using the LAQN online measurement archive (www.londonair.org.uk), a significant difference in concentration means was found between site types. Site type contrasts were also identified in diurnal fluctuations where the magnitude of NO_x_ increase (indicative of primary vehicle emissions) was greater at roadside sites than at urban background locations.

A large carriageway was approximately 1.5 km west of Urban Background Site B with a busy residential road also running 300 m east of the site. These nearby roadways yielded the elevated PNC and NO_x_ levels when data were analysed according to wind direction. Urban Background Site D was situated 400 m southeast of a major carriageway which likely caused the observed increase in PNC from this wind direction. Significantly lower primary particle and gaseous pollutant concentrations were measured at Urban Background Site C where the site was located on the roof of a three storey primary school. Although it is useful to note that two multi-lane carriageways, running east-west, are located 200 m north and south of the site.

The contribution of vehicle emissions to PM mass concentrations (TEOM PM_10_×1.3: Sites A-E; FDMS PM_2.5_: Sites F and G) was quantitatively evaluated at all sites using Pearson correlations between 15 minute resolved PM mass and NO_x_ concentrations for the total two week sampling period [26]–[28]. Roadside Sites F and G, as expected, exhibited the strongest significant (p<0.0001) relationships (Site F: r^2^ = 0.81, n = 1274; Site G: r^2^ = 0.81, n = 1313) and the greatest contribution of vehicle derived PM. At Roadside Site E, the relationship between PM_10_ and NO_x_ was decreased compared to Sites F and G (r^2^ = 0.27, n = 1220). Correlations were weaker but still significant for all urban background locations (Site B: r^2^ = 0.58, n = 1193; Site C: r^2^ = 0.07, n = 1475; Site D: r^2^ = 0.62, n = 1540) and the suburban site (Site A: r^2^ = 0.34, n = 1065); the lowest PM_10_-NO_x_ association for Urban Background Site C was attributable to its roof-top location. Despite proximity of this site to a major carriageway, air quality was likely affected by a vertical gradient of PNC and NO_x_ concentrations [29]. Therefore, regardless of the site type, sampled PM was influenced by vehicular sources to some degree.

It can be further shown that as the variance in PM mass concentration explained by NO_x_ increased, the concentration of Fe (p = 0.004), Cu (p = 0.022), and Ba (p = 0.007) also increased proportionally; this increase was not found to be statistically significant for any of the other metals measured. Fe, Cu and Ba were thus likely reflective of traffic derived emissions from brake wear [30] which were evident at all seven sampling sites.

### Size-fractionated trace metal concentrations

**Figure 2:**
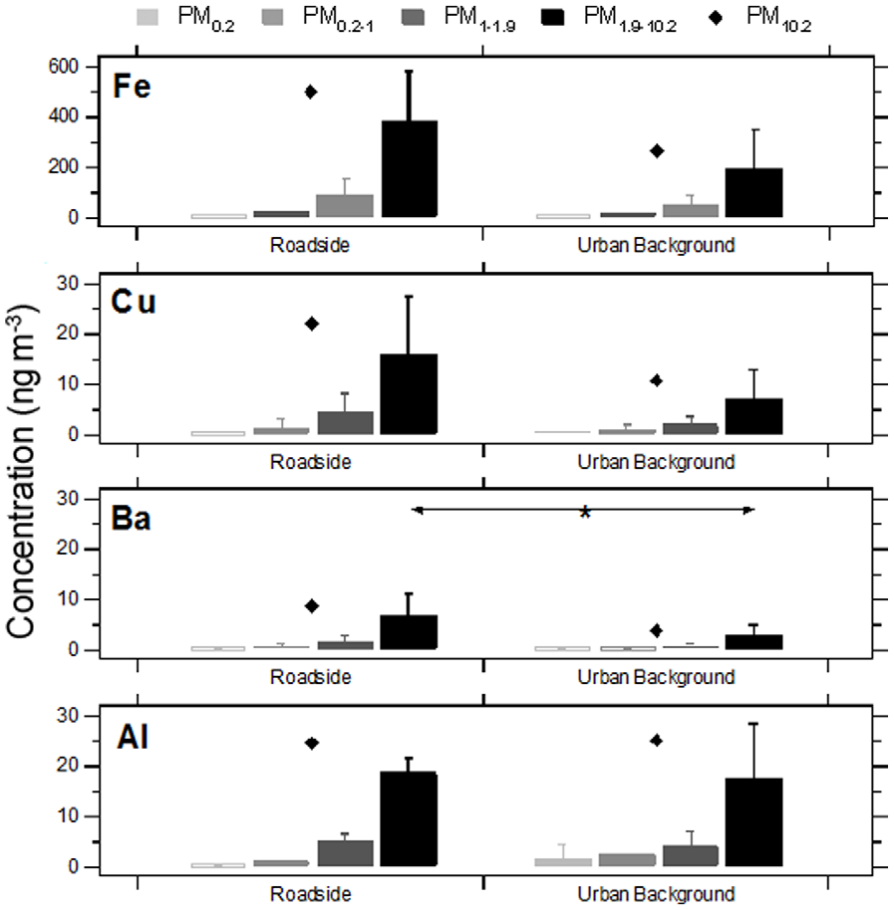
Mean size-fractionated PM trace metal (Fe, Cu, Ba, Al) concentrations at primary schools grouped by roadside and urban background site types. Each bar represents the mean and associated standard deviation. Diamond markers indicate the mean total PM_10.2_ trace metal concentration for each site. Comparison of roadside and urban background means between PM size fractions was performed using a one-way ANOVA with post-hoc testing using the Tukey HSD test. Significant contrast (p<0.05) are noted.

**Figure 3:**
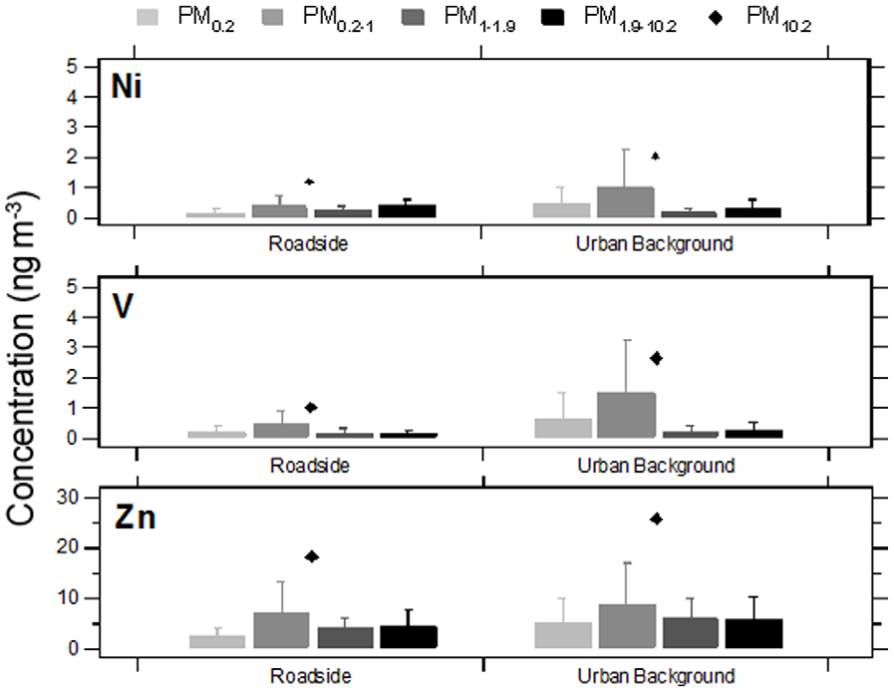
Mean size-fractionated PM trace metal (Ni, V, Zn) concentrations at primary school grouped by roadside and urban background site types. See the Figure 2 legend for a description of marker and statistical details.

**Figure 4:**
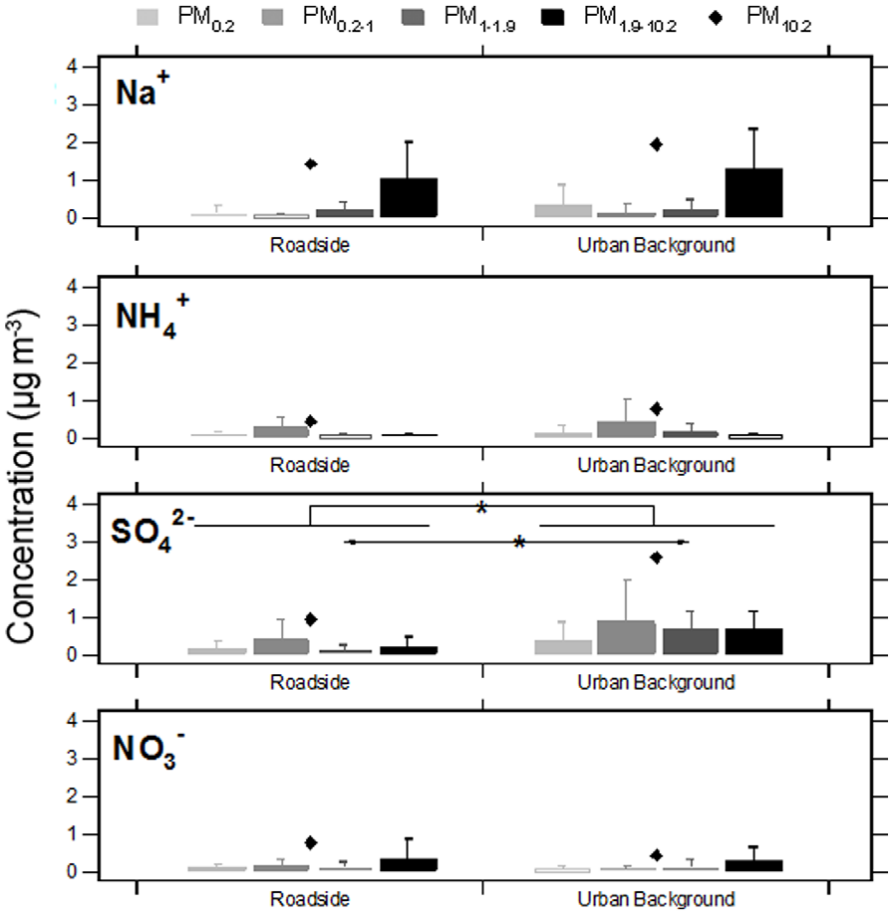
Mean size-fractionated PM soluble inorganic constituent concentrations at primary school grouped by roadside and urban background site types. See the Figure 2 legend for a description of marker and statistical details.

Individual schools were grouped by urban background and roadside classifications and contrasts in mean size-fractionated trace metal concentrations were evaluated. Consistent with the NO_x_ and PM_10_ mass concentration results, no significant differences were identified in trace metal concentrations for each of the measured size fractions between the two site types (Figure 2 and Figure 3); the only exception to this was PM_1.9–10_ Ba where concentrations were significantly greater at the roadside site schools. For the roadside site school grouping, Ba, Fe, Cu and Al concentrations were significantly elevated in the PM_1.9–10_ size fraction in comparison to other size fractions. Although, the increase of these trace metals was not significant for the urban background site group, a similar enrichment pattern was also observed. Moderate but not statistically significant enrichment of Ni, V and Zn in the PM_0.2–1_ size fraction was identified at roadside and urban background sites (Figure 3). As shown in Figure 4, these PM_0.2–1_ modal increases were accompanied by elevations in NH_4_^+^ and SO_4_^2−^ concentrations, most probably associated with long-range atmospheric transport processes. Data from individual sites appear in Figures S1, S2 and S3.

Although homogeneity of the size-fractionated trace metal profiles found at the two site types likely reflects the restricted, sequential sampling periods, which were subject to short-term meteorological effects, results confirm that both roadside and urban background schools were influenced by the same panel of sources. To identify these contributing sources common to both site types, bi-variate trace metal concentrations correlations were performed on each of the collected size-fractionated.

A strong relationship (p<0.01) was observed between Cu and Ba reflecting their common source in brake dust for all size-fractions other than PM_0.2_. All size-fractions of Ba and Cu were also well correlated with Fe (p<0.01); PM_0.2_ was again the exception for Cu-Fe correlations. Fe, Ba, and Cu are all derived from non-tailpipe traffic emissions and this common source was reflected in the strong positive correlations between species. Gietl et al. [30] and Birmili et al. [31] reported that these three trace metals all result from single uniform abrasive vehicular emissions, primarily brake pad debris; similarities were identified in temporal fluctuations, with the strongest linearity between these metal concentrations observed in the coarse mode.

Ni and V exhibited a strong association for all sample size-fractions (p<0.01). A relationship was found for Ni and V with Zn for all size-fractions (p<0.01) with the exception of V-Zn for PM_0.2_. Ni, V and Zn may be derived from both non-tailpipe vehicle [32]–[35] and oil combustion emissions [36]–[38]. A significant relationship between Zn with Fe and Cu (p<0.05) was observed for PM_0.2–1_ (p<0.01) and PM_1.9–10.2_. Ni and V were also both correlated with Fe for PM_0.2_ (p<0.01) and to a lesser extent with PM_0.2–1_ (p<0.05). However, for total PM_10.2_ mass, no correlation existed between Ni (r = 0.47, p = 0.09) and V (r = 0.36, p = 0.21) with Fe. V also lacked any correlation with Cu and Ba. Although a relationship was established between Ni, V, and Zn and known vehicular trace metal markers, episodic concentrations exclusive to these trace metals (90% percentile) were measured during only two of the site visits (Urban Background Site B week 2 and Site D week 1). Occurrence of elevated concentrations in the absence of elevation in the other metals suggests another potential source type. The United Kingdom National Atmospheric Emissions Inventory reports Ni and V are primary emitted from industrial fuel oil combustion and shipping sources, which do not have a local source in the Greater London Urban Area. Enrichment of Ni and V was noted in a similar PM size-fraction (0.1–1 µm) in Helsinki, Finland where these species were attributed to oil combustion [39]. Similarly, Allen et al. [17] noted that size-fractionated Zn and Ni sampled in Birmingham, United Kingdom had the greatest concentrations in the accumulation mode and were thought to be the result of long range transported combustion sources. Moreover, Ni (r = 0.63; p = 0.02) and V (r = 0.66; p = 0.01) concentrations were significantly correlated with SO_4_^2−^ concentrations. Gaseous SO_2_ accompanies Ni and V in from shipping emissions [36], [40]–[42] and from other fuel oil combustion sources, and following long range transport, atmospheric processing would result in SO_2_ oxidation to particulate SO_4_^2−^.

### Particulate oxidative potential

**Figure 5:**
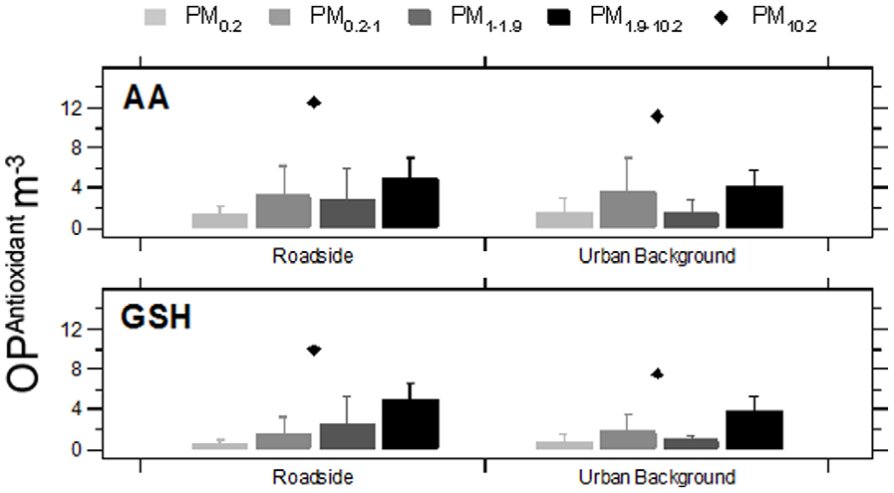
Mean size-fractionated PM ascorbate and glutathione related oxidative potential at primary school grouped by roadside and urban background site types. See the Figure 2 legend for a description of marker and statistical details.

Previous studies have indicated an increment in PM OP at high-traffic versus urban background locations [43] associated with elevated redox active Fe and Cu content [44]. However, no robust site classification-dependent contrasts were found for either of the OP metrics in any of the size fractions measured (Figure 5). When variation across individual sites was considered, only Roadside Site F, which showed a strong traffic influence upon trace metal concentrations also exhibited the highest OP in both tests (Figure S4).

**Figure 6:**
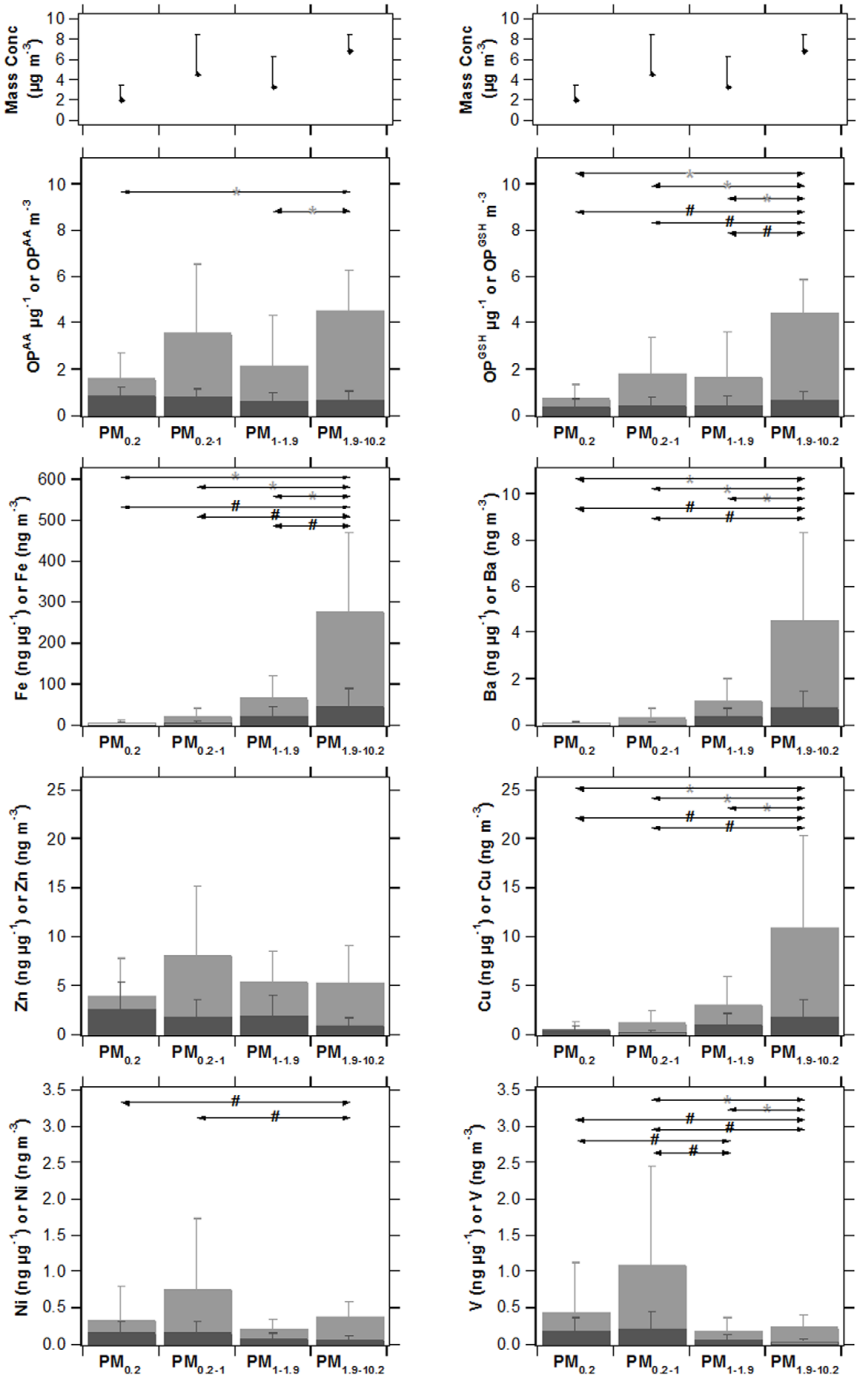
Size-fractionated PM mass concentration, OP^AA^, OP^GSH^, and trace metals levels per unit mass and unit volume. Each bar represents the mean across the seven primary school sampling sites with the associated standard deviation. Dark and light grey column indicate measurements per unit mass and volume, respectively. Comparison of means between the PM size fractions was performed using a one-way ANOVA with post-hoc testing using the Tukey HSD test. Significant contrast (p<0.05) are noted for measurements per unit mass (#) and per unit volume (*).

As common sources were found to influence both the roadside and urban background school sites, all seven sites were aggregated and the mean size-fractionated OP response was evaluated. Distinct size fraction dependent contrasts were apparent in the data expressed per cubic metre, with highest PM OP associated with the coarse fraction. Some contrast in OP^GSH^ µg^−1^ was found across the four PM size-fractions sampled but only a significant contrast between PM_0.2_ (0.38±0.16 µg^−1^) and PM_1.9–10.2_ (0.69±0.27 µg^−1^) (Figure 6). Levels of the OP^GSH^ µg^−1^ metric increased with PM size and this occurred in parallel with measured metal species known to have a traffic origin, with concentrations of Fe, Cu, and Ba (ng µg^−1^) all enriched in the larger size fraction. Significant increases of these metals in larger size fractions, however, did not yield significant increases in OP^AA^ µg^−1^ (α = 0.05); with no differences in this metric were identifiable between the four size fractions sampled. Homogeneity in OP^AA^ µg^−1^ levels suggests a counteracting influence of components enriched in the smaller size fractions, possible Zn, Ni and V (Figure 6), but possibly also including trace organic compounds, or an effect of greater surface area per unit mass in smaller particles.

To further examine the relationship of OP metrics variance and trace metal concentrations, bi-variate correlation analysis was performed. A statistically significant correlation was observed for not only OP^GSH^ µg^−1^ and Fe (ng µg^−1^; r^2^ = 0.38, p = 0.02) but also OP^GSH^ µg^−1^ and Cu (ng µg^−1^; r^2^ = 0.31, p = 0.04). It is also useful to recall that both these redox active trace metals expressed per unit mass were internally correlated (r = 0.91, p<0.0001), suggestive of a common source. The strength of the relationship between OP^AA^ µg^−1^ and individual trace metals was in contrast only found to be significant for Ni (ng µg^−1^; r^2^ = 0.41, p = 0.01) and V (ng µg^−1^; r^2^ = 0.37, p = 0.02). This difference in the potential predictors of these antioxidant metrics was expected as no relationship existed between OP^AA^ µg^−1^ and OP^GSH^ µg^−1^ (r = 0.16, p = 0.59). This examination of antioxidant trends across PM size fractions thus suggested that AA and GSH depletion are sensitive to different panels of PM constituents, with a relationship across the measured size fractions between the GSH dependent OP metric and traffic related trace metals. Although total particulate oxidative burden as indicated by AA depletion was influenced by traffic derived metals, other sources responsible for elevated ambient Ni and V concentrations were predictive of this antioxidant's depletion.

The combined effects of what appeared to be two distinct sources (abrasive vehicular and a long range transported fuel oil combustion emissions) at these London sampling sites was investigated through linear regression modelling. Full details appear in Table S1. Trace metal factors represented the best determinants of the associated PM_10.2_ OP, consistent with previous studies [20], [45], [46]. The multivariate models constructed to predict PM_10.2_ OP variance for both antioxidant metrics included iron as a factor (Table 2). PM-induced GSH oxidation in the RTLF model was sensitive to a source characteristic of Fe (and Cu), thus likely related to traffic derived emissions. Variance in the OP measured via AA depletion was best described by Fe and Zn concentrations. Both of these trace metals are primarily the result of non-tail pipe vehicle emissions; however, from the strong relationship of Zn with Ni (ng µg^−1^; r = 0.76, p<0.0001) and V (ng µg^−1^; r = 0.71, p<0.0001) coupled with the strength of the bi-variate correlation results of OP^AA^ µg^−1^ – Ni (ng µg^−1^) and OP^AA^ µg^−1^ – V (ng µg^−1^), it was likely that AA depletion measured in this London study was also sensitive to a long range fuel oil combustion source. When PM_10.2_ mass was forced into these model as an explanatory variable, the model's ability to predict OP variance over the trace metal variables alone did not increase. Moreover, no association was found between PM_10.2_ mass and AA or GSH related OP µg^−1^. It should be emphasised that elucidating the trace metals associated with OP^AA^ µg^−1^ and OP^GSH^ µg^−1^ metrics in the multi-variate regression method was ultimately limited by the statistical method used as the co-linearity of trace metals confounds results. Identification of the independent contribution of individual trace metals to OP was therefore limited if a common source origin existed.

**Table 2:** Parameters of multi-variate linear regression models to explain PM_10.2_ oxidative potential metric variance.

Metric	Metal	Explanatory Variable			Model	
		B	SE	p-value	R^2^	p-value
OP^AA^ ug^−1^	Fe	0.01	0.004	0.03	0.52	0.02
	Zn	0.18	0.07	0.03		
OP^GSH^ ug^−1^	Fe	0.08	0.02	-	0.38	0.02

Only PM constituents with intrinsic redox activity, including quinones and redox active metals, drive antioxidant depletion in the synthetic RTLF chemical model. Certain redox active trace metals were identified as significantly associated with the OP metrics and may be directly responsible for AA and GSH depletion. The current study did not measure the quinone composition of the sampled PM; however, non-redox active metals found to be predictive of OP via uni- and multi-variate regression analysis likely represent surrogate measures of constituents with intrinsic and non-latent redox active properties which were not quantified. Elucidating why individual RTLF antioxidants are sensitive to heterogeneous PM constituents is the subject of future studies being conducted currently and involving more extensive PM chemical characterisation.

## Conclusions

This study demonstrates both the feasibility of performing a detailed air quality assessment, including compositional analysis of size fractionated PM and assessment of PM OP, at primary schools in London. The depth of analysis that can be applied to this data set is somewhat limited by the small number of visits performed at each location, but it is clear that coarser PM fractions contribute significantly to the OP in the ambient PM and should not therefore be excluded in consideration of PM health impacts. The regression analysis performed demonstrated that select redox active and non-active metals are better able to explain variance in PM OP than the bulk PM mass concentration. A consistent trend across all sites of elevated Fe mass concentrations in the coarse mode particles and strong correlations with other abrasive traffic markers (Cu and Ba) suggest the Fe associated with increased PM OP is related to vehicle emissions.

This study did not demonstrate an increment in PM OP at roadside school sites compared to those situated away from major roads. However, the locations of all schools examined were found to experience periods of high traffic influence (stop-go and idling) at the start and end of the school day and were subject to the advected traffic pollution. Therefore, regardless of the school site in London, there will be a traffic emission contribution to ambient PM. These emissions will increase the concentration of redox cycling trace metals which in turn cause a greater particulate oxidative burden.

## Supporting Information

Figure S1 Mean size-fractionated PM trace metal (Fe, Cu, Ba, Al) concentrations at primary school sampling sites. Each bar represents the mean of two filter samples per site visit with the associated standard deviation. Diamond markers indicate the mean total PM_10.2_ trace metal concentration for each site.
(TIF)

Figure S2 Mean size-fractionated PM trace metal (Ni, V, Zn) concentrations at primary school sampling sites. Each bar represents the mean of two filter samples per site visit with the associated standard deviation. Diamond markers indicate the mean total PM_10.2_ trace metal concentration for each site.
(TIF)

Figure S3 Mean size-fractionated PM soluble inorganic constituent concentrations at primary school sampling sites. Each bar represents the mean of two filter samples per site visit with the associated standard deviation. Diamond markers indicate the mean total PM_10.2_ soluble inorganic constituent concentration for each site.
(TIF)

Figure S4 Mean size-fractionated PM ascorbate and glutathione related oxidative potential at primary school sampling sites. Each bar represents the mean of two filter samples per site visit with the associated standard deviation. Diamond markers indicate the mean total PM_10.2_ oxidative potential for each site.
(TIF)

Table S1 Bivariate correlation analysis for OP^AA^, OP^GSH^, mass concentration and metal variables expressed per unit volume in the top triangle (blue) and per unit mass in the bottom triangle (green) for PM_10.2_ and individual PM size fractions (N = 14). Correlations with p values less than 0.01 and 0.05 are highlighted in yellow and orange, respectively.
(DOC)
