# Supplementary figures and images for: Increased Oxidative Burden Associated with Traffic Component of Ambient Particulate Matter at Roadside and Urban Background Schools Sites in London
Source: PLoS One. 2011 Jul 27;6(7):e21961. doi: 10.1371/journal.pone.0021961 (PMC3144873; doi:10.1371/journal.pone.0021961)

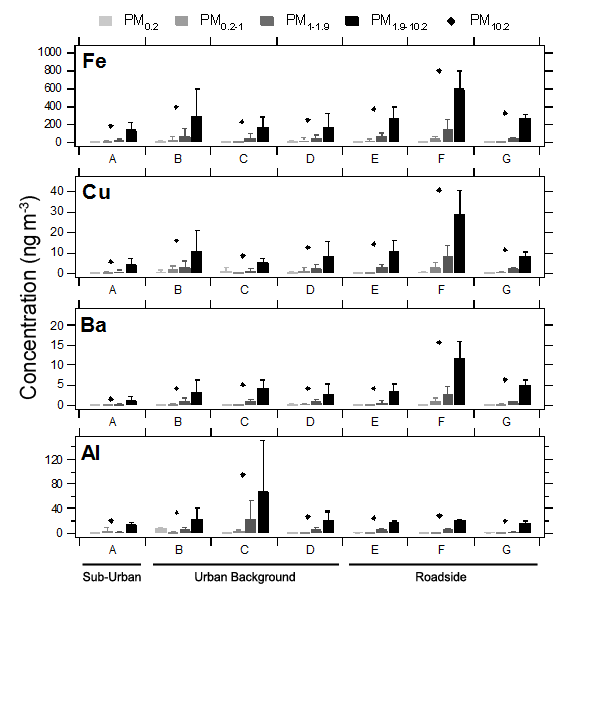

Supplement: Figure S1 — Mean size-fractionated PM trace metal (Fe, Cu, Ba, Al) concentrations at primary school sampling sites. Each bar represents the mean of two filter samples per site visit with the associated standard deviation. Diamond markers indicate the mean total PM10.2 trace metal concentration for each site. (TIF) [file pone.0021961.s001.tif]

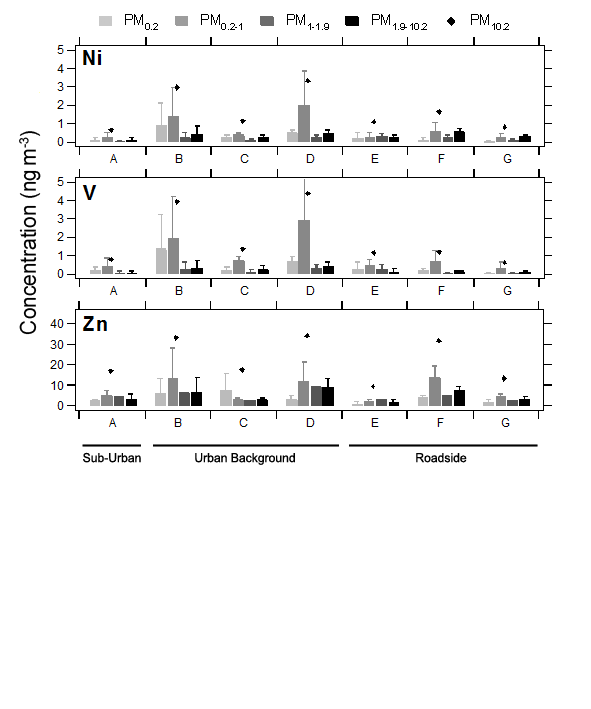

Supplement: Figure S2 — Mean size-fractionated PM trace metal (Ni, V, Zn) concentrations at primary school sampling sites. Each bar represents the mean of two filter samples per site visit with the associated standard deviation. Diamond markers indicate the mean total PM10.2 trace metal concentration for each site. (TIF) [file pone.0021961.s002.tif]

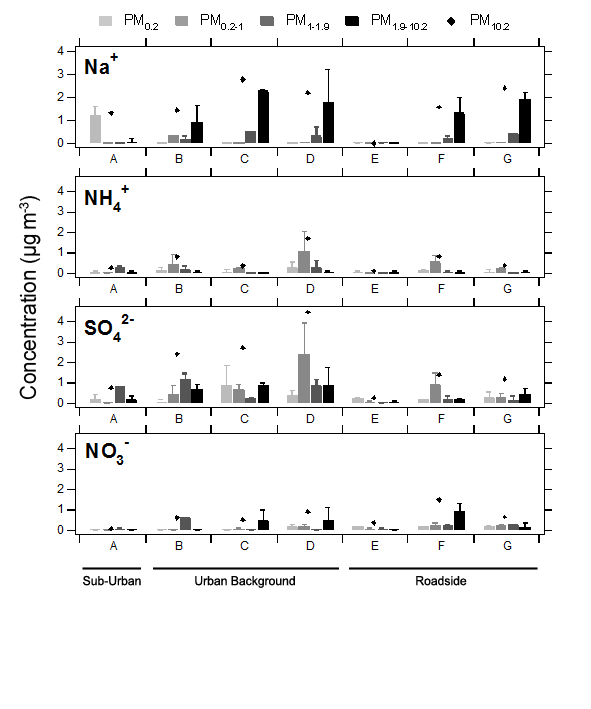

Supplement: Figure S3 — Mean size-fractionated PM soluble inorganic constituent concentrations at primary school sampling sites. Each bar represents the mean of two filter samples per site visit with the associated standard deviation. Diamond markers indicate the mean total PM10.2 soluble inorganic constituent concentration for each site. (TIF) [file pone.0021961.s003.tif]

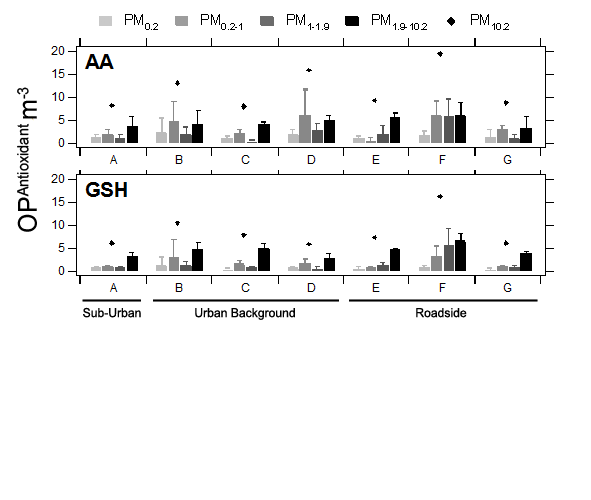

Supplement: Figure S4 — Mean size-fractionated PM ascorbate and glutathione related oxidative potential at primary school sampling sites. Each bar represents the mean of two filter samples per site visit with the associated standard deviation. Diamond markers indicate the mean total PM10.2 oxidative potential for each site. (TIF) [file pone.0021961.s004.tif]
