# Supplementary material for: Increased Oxidative Burden Associated with Traffic Component of Ambient Particulate Matter at Roadside and Urban Background Schools Sites in London
Source: PLoS One. 2011 Jul 27;6(7):e21961. doi: 10.1371/journal.pone.0021961 (PMC3144873; doi:10.1371/journal.pone.0021961)
Supplement: Table S1 — Bivariate correlation analysis for OPAA, OPGSH, mass concentration and metal variables expressed per unit volume in the top triangle (blue) and per unit mass in the bottom triangle (green) for PM10.2 and individual PM size fractions (N = 14). Correlations with p values less than 0.01 and 0.05 are highlighted in yellow and orange, respectively. (DOC) [file pone.0021961.s005.doc]

**Table S1.** Bivariate correlation analysis for OP^AA^, OP^GSH^, mass concentration and metal variables expressed per unit volume in the top triangle (blue) and per unit mass in the bottom triangle (green) for PM_10.2_ and individual PM size fractions (N=14). Correlations with p values less than 0.01 and 0.05 are highlighted in yellow and orange, respectively.

| **PM_10.2_** | **OP^AA^** | **OP^GSH^** | **Mass Conc** | **Fe** | **Cu** | **Ba** | **Al** | **Zn** | **V** | **Ni** |
| --- | --- | --- | --- | --- | --- | --- | --- | --- | --- | --- |
| **OP^AA^** |  | 0.77 | 0.92 | 0.78 | 0.76 | 0.68 | 0.1 | 0.84 | 0.71 | 0.79 |
|  |  | *0.00* | *0.00* | *0.00* | *0.00* | *0.01* | *0.74* | *0.00* | *0.00* | *0.00* |
| **OP^GSH^** | 0.16 |  | 0.91 | 0.87 | 0.82 | 0.78 | 0.24 | 0.62 | 0.35 | 0.45 |
|  | *0.59* |  | *0.00* | *0.00* | *0.00* | *0.00* | *0.41* | *0.02* | *0.21* | *0.11* |
| **Mass** | 0.46 | 0.41 |  | 0.81 | 0.79 | 0.75 | 0.28 | 0.77 | 0.58 | 0.66 |
|  | *0.10* | *0.15* |  | *0.00* | *0.00* | *0.00* | *0.33* | *0.00* | *0.03* | *0.01* |
| **Fe** | 0.48 | 0.62 | 0.34 |  | 0.96 | 0.93 | 0.16 | 0.57 | 0.36 | 0.47 |
|  | *0.08* | *0.02* | *0.23* |  | *0.00* | *0.00* | *0.60* | *0.03* | *0.21* | *0.09* |
| **Cu** | 0.45 | 0.56 | 0.36 | 0.91 |  | 0.94 | 0.01 | 0.58 | 0.31 | 0.45 |
|  | *0.11* | *0.04* | *0.21* | *0.00* |  | *0.00* | *0.97* | *0.03* | *0.28* | *0.11* |
| **Ba** | 0.32 | 0.48 | 0.42 | 0.85 | 0.86 |  | 0.17 | 0.42 | 0.14 | 0.27 |
|  | *0.26* | *0.08* | *0.13* | *0.00* | *0.00* |  | *0.56* | *0.13* | *0.63* | *0.34* |
| **Al** | -0.21 | 0.10 | 0.33 | 0.02 | -0.19 | 0.08 |  | 0.01 | 0.18 | 0.13 |
|  | *0.48* | *0.74* | *0.24* | *0.95* | *0.51* | *0.80* |  | *0.97* | *0.53* | *0.67* |
| **Zn** | 0.51 | -0.05 | 0.40 | -0.05 | 0.13 | -0.06 | -0.32 |  | 0.86 | 0.91 |
|  | *0.06* | *0.87* | *0.16* | *0.86* | *0.67* | *0.83* | *0.27* |  | *0.00* | *0.00* |
| **V** | 0.61 | -0.17 | 0.46 | 0.10 | 0.12 | -0.08 | 0.03 | 0.71 |  | 0.99 |
|  | *0.02* | *0.56* | *0.10* | *0.74* | *0.69* | *0.77* | *0.93* | *0.00* |  | *0.00* |
| **Ni** | 0.64 | -0.13 | 0.45 | 0.17 | 0.23 | 0.02 | -0.08 | 0.76 | 0.98 |  |
|  | *0.01* | *0.65* | *0.11* | *0.56* | *0.42* | *0.95* | *0.77* | *0.00* | *0.00* |  |

** It is useful to note that the strong association between (1) Fe, Cu and Ba and (2) Ni and V for PM_10.2_ is predominantly driven by the PM_1.9-10.2_ and PM_0.2-1_ fractions, respectively, where all these metals were enriched.**

| **PM_0.2_** | **OP^AA^** | **OP^GSH^** | **Mass Conc** | **Fe** | **Cu** | **Ba** | **Zn** | **V** | **Ni** |
| --- | --- | --- | --- | --- | --- | --- | --- | --- | --- |
| **OP^AA^** |  | 0.93 | 0.94 | 0.74 | 0.19 | 0.41 | 0.49 | 0.83 | 0.81 |
|  |  | *0.00* | *0.00* | *0.00* | *0.52* | *0.15* | *0.08* | *0.00* | *0.00* |
| **OP^GSH^** | 0.74 |  | 0.91 | 0.69 | 0.19 | 0.27 | 0.53 | 0.90 | 0.89 |
|  | *0.00* |  | *0.00* | *0.01* | *0.51* | *0.36* | *0.05* | *0.00* | *0.00* |
| **Mass** | -0.24 | -0.01 |  | 0.73 | 0.16 | 0.49 | 0.45 | 0.77 | 0.75 |
|  | *0.41* | *0.98* |  | *0.00* | *0.58* | *0.08* | *0.11* | *0.00* | *0.00* |
| **Fe** | 0.49 | 0.45 | -0.16 |  | 0.41 | 0.81 | 0.50 | 0.70 | 0.70 |
|  | *0.08* | *0.11* | *0.59* |  | *0.16* | *0.00* | *0.09* | *0.01* | *0.01* |
| **Cu** | 0.18 | 0.17 | -0.24 | 0.65 |  | 0.29 | 0.90 | 0.22 | 0.34 |
|  | *0.54* | *0.56* | *0.41* | *0.01* |  | *0.31* | *0.00* | *0.46* | *0.24* |
| **Ba** | -0.18 | -0.35 | -0.06 | 0.44 | 0.16 |  | 0.17 | 0.28 | 0.27 |
|  | *0.55* | *0.21* | *0.85* | *0.12* | *0.58* |  | *0.55* | *0.34* | *0.34* |
| **Zn** | 0.18 | 0.21 | -0.22 | 0.62 | 0.99 | 0.08 |  | 0.52 | 0.63 |
|  | *0.54* | *0.47* | *0.45* | *0.02* | *0.00* | *0.78* |  | *0.05* | *0.02* |
| **V** | -0.02 | 0.28 | 0.45 | 0.08 | -0.10 | 0.00 | -0.06 |  | 0.99 |
|  | *0.94* | *0.33* | *0.11* | *0.77* | *0.74* | *0.99* | *0.85* |  | *0.00* |
| **Ni** | 0.08 | 0.32 | 0.16 | 0.45 | 0.57 | 0.08 | 0.61 | 0.75 |  |
|  | *0.78* | *0.26* | *0.58* | *0.10* | *0.03* | *0.78* | *0.02* | *0.00* |  |

| **PM_0.2-1_** | **OP^AA^** | **OP^GSH^** | **Mass Conc** | **Fe** | **Cu** | **Ba** | **Zn** | **V** | **Ni** |
| --- | --- | --- | --- | --- | --- | --- | --- | --- | --- |
| **OP^AA^** |  | 0.79 | 0.94 | 0.80 | 0.85 | 0.73 | 0.93 | 0.82 | 0.85 |
|  |  | *0.00* | *0.00* | *0.00* | *0.00* | *0.00* | *0.00* | *0.00* | *0.00* |
| **OP^GSH^** | 0.62 |  | 0.84 | 0.86 | 0.87 | 0.72 | 0.88 | 0.56 | 0.60 |
|  | *0.02* |  | *0.00* | *0.00* | *0.00* | *0.00* | *0.00* | *0.04* | *0.02* |
| **Mass** | -0.28 | -0.21 |  | 0.90 | 0.95 | 0.84 | 0.92 | 0.73 | 0.78 |
|  | *0.38* | *0.52* |  | *0.00* | *0.00* | *0.00* | *0.00* | *0.00* | *0.00* |
| **Fe** | -0.11 | 0.21 | 0.26 |  | 0.90 | 0.79 | 0.83 | 0.60 | 0.64 |
|  | *0.71* | *0.46* | *0.42* |  | *0.00* | *0.00* | *0.00* | *0.02* | *0.01* |
| **Cu** | 0.37 | 0.19 | 0.40 | 0.06 |  | 0.94 | 0.86 | 0.51 | 0.57 |
|  | *0.20* | *0.51* | *0.20* | *0.85* |  | *0.00* | *0.00* | *0.06* | *0.03* |
| **Ba** | 0.45 | 0.29 | 0.26 | -0.03 | 0.75 |  | 0.66 | 0.29 | 0.34 |
|  | *0.11* | *0.32* | *0.42* | *0.92* | *0.00* |  | *0.01* | *0.32* | *0.23* |
| **Zn** | 0.47 | 0.22 | -0.17 | -0.22 | 0.51 | 0.14 |  | 0.80 | 0.83 |
|  | *0.09* | *0.45* | *0.60* | *0.44* | *0.06* | *0.62* |  | *0.00* | *0.00* |
| **V** | 0.14 | 0.15 | 0.23 | 0.04 | -0.33 | -0.29 | -0.08 |  | 1.00 |
|  | *0.62* | *0.60* | *0.48* | *0.90* | *0.25* | *0.31* | *0.79* |  | *0.00* |
| **Ni** | 0.16 | 0.08 | 0.31 | 0.03 | -0.27 | -0.28 | 0.02 | 0.97 |  |
|  | *0.59* | *0.78* | *0.33* | *0.92* | *0.36* | *0.34* | *0.93* | *0.00* |  |

| **PM_1-1.9_** | **OP^AA^** | **OP^GSH^** | **Mass Conc** | **Fe** | **Cu** | **Ba** | **Zn** | **V** | **Ni** |
| --- | --- | --- | --- | --- | --- | --- | --- | --- | --- |
| **OP^AA^** |  | 0.89 | 0.91 | 0.36 | 0.42 | 0.36 | 0.37 | 0.32 | 0.38 |
|  |  | *0.00* | *0.00* | *0.21* | *0.14* | *0.20* | *0.19* | *0.27* | *0.18* |
| **OP^GSH^** | 0.15 |  | 0.97 | 0.34 | 0.39 | 0.37 | 0.03 | -0.06 | 0.11 |
|  | *0.61* |  | *0.00* | *0.24* | *0.17* | *0.19* | *0.91* | *0.84* | *0.71* |
| **Mass** | 0.09 | 0.27 |  | 0.19 | 0.23 | 0.22 | 0.08 | 0.03 | 0.10 |
|  | *0.75* | *0.36* |  | *0.50* | *0.43* | *0.44* | *0.78* | *0.93* | *0.75* |
| **Fe** | 0.35 | 0.63 | -0.17 |  | 0.96 | 0.94 | 0.31 | 0.33 | 0.64 |
|  | *0.22* | *0.02* | *0.57* |  | *0.00* | *0.00* | *0.28* | *0.24* | *0.01* |
| **Cu** | 0.42 | 0.67 | -0.15 | 0.96 |  | 0.96 | 0.30 | 0.24 | 0.60 |
|  | *0.14* | *0.01* | *0.61* | *0.00* |  | *0.00* | *0.30* | *0.42* | *0.02* |
| **Ba** | 0.30 | 0.58 | -0.14 | 0.92 | 0.94 |  | 0.26 | 0.17 | 0.50 |
|  | *0.30* | *0.03* | *0.63* | *0.00* | *0.00* |  | *0.37* | *0.56* | *0.07* |
| **Zn** | 0.54 | -0.33 | -0.38 | 0.10 | 0.11 | 0.12 |  | 0.80 | 0.66 |
|  | *0.05* | *0.25* | *0.18* | *0.73* | *0.71* | *0.70* |  | *0.00* | *0.01* |
| **V** | 0.64 | -0.32 | -0.17 | 0.19 | 0.12 | 0.10 | 0.71 |  | 0.82 |
|  | *0.01* | *0.26* | *0.57* | *0.52* | *0.69* | *0.74* | *0.00* |  | *0.00* |
| **Ni** | 0.44 | 0.08 | -0.38 | 0.51 | 0.50 | 0.44 | 0.49 | 0.66 |  |
|  | *0.12* | *0.78* | *0.19* | *0.06* | *0.07* | *0.12* | *0.08* | *0.01* |  |

| **PM_1.9-10.2_** | **OP^AA^** | **OP^GSH^** | **Mass Conc** | **Fe** | **Cu** | **Ba** | **Zn** | **V** | **Ni** |
| --- | --- | --- | --- | --- | --- | --- | --- | --- | --- |
| **OP^AA^** |  | 0.66 | -0.16 | 0.73 | 0.69 | 0.62 | 0.49 | 0.49 | 0.70 |
|  |  | *0.01* | *0.59* | *0.00* | *0.01* | *0.02* | *0.07* | *0.08* | *0.01* |
| **OP^GSH^** | 0.77 |  | 0.05 | 0.86 | 0.81 | 0.84 | 0.34 | 0.25 | 0.64 |
|  | *0.00* |  | *0.85* | *0.00* | *0.00* | *0.00* | *0.24* | *0.38* | *0.01* |
| **Mass** | -0.42 | -0.37 |  | -0.31 | -0.40 | -0.17 | -0.33 | 0.04 | -0.31 |
|  | *0.13* | *0.19* |  | *0.27* | *0.16* | *0.55* | *0.25* | *0.88* | *0.28* |
| **Fe** | 0.80 | 0.96 | -0.39 |  | 0.96 | 0.93 | 0.54 | 0.39 | 0.86 |
|  | *0.00* | *0.00* | *0.16* |  | *0.00* | *0.00* | *0.05* | *0.17* | *0.00* |
| **Cu** | 0.78 | 0.95 | -0.42 | 0.97 |  | 0.93 | 0.56 | 0.30 | 0.83 |
|  | *0.00* | *0.00* | *0.14* | *0.00* |  | *0.00* | *0.04* | *0.30* | *0.00* |
| **Ba** | 0.69 | 0.91 | -0.30 | 0.94 | 0.96 |  | 0.42 | 0.23 | 0.77 |
|  | *0.01* | *0.00* | *0.29* | *0.00* | *0.00* |  | *0.13* | *0.43* | *0.00* |
| **Zn** | 0.62 | 0.53 | -0.33 | 0.61 | 0.62 | 0.51 |  | 0.71 | 0.82 |
|  | *0.02* | *0.05* | *0.25* | *0.02* | *0.02* | *0.06* |  | *0.00* | *0.00* |
| **V** | 0.59 | 0.33 | -0.14 | 0.45 | 0.40 | 0.27 | 0.80 |  | 0.72 |
|  | *0.02* | *0.25* | *0.62* | *0.11* | *0.16* | *0.34* | *0.00* |  | *0.00* |
| **Ni** | 0.79 | 0.78 | -0.40 | 0.87 | 0.85 | 0.79 | 0.86 | 0.78 |  |
|  | *0.00* | *0.00* | *0.16* | *0.00* | *0.00* | *0.00* | *0.00* | *0.00* |  |
